# A Series of PSMA-Targeted Near-Infrared Fluorescent Imaging Agents

**DOI:** 10.3390/biom12030405

**Published:** 2022-03-05

**Authors:** Ying Chen, Il Minn, Steven P. Rowe, Alla Lisok, Samit Chatterjee, Mary Brummet, Sangeeta Ray Banerjee, Ronnie C. Mease, Martin G. Pomper

**Affiliations:** The Russell H. Morgan Department of Radiology and Radiological Science, Johns Hopkins University School of Medicine, Baltimore, MD 21287, USA; ychen95jhmi@gmail.com (Y.C.); iminn1@jhmi.edu (I.M.); srowe8@jhmi.edu (S.P.R.); alisok1@jhmi.edu (A.L.); samitchat@gmail.com (S.C.); mbrummet@jhmi.edu (M.B.); sray9@jhmi.edu (S.R.B.); rmease1@jhmi.edu (R.C.M.)

**Keywords:** NIRF, molecular imaging, fluorescence-guided surgery, prostate-specific membrane antigen, DyLight800

## Abstract

We have synthesized a series of 10 new, PSMA-targeted, near-infrared imaging agents intended for use in vivo for fluorescence-guided surgery (FGS). Compounds were synthesized from the commercially available amine-reactive active NHS ester of DyLight800. We altered the linker between the PSMA-targeting urea moiety and the fluorophore with a view to improve the pharmacokinetics. Chemical yields for the conjugates ranged from 51% to 86%. The *K*_i_ values ranged from 0.10 to 2.19 nM. Inclusion of an *N*-bromobenzyl substituent at the ε-amino group of lysine enhanced PSMA^+^ PIP tumor uptake, as did hydrophilic substituents within the linker. The presence of a polyethylene glycol chain within the linker markedly decreased renal uptake. In particular, DyLight800-**10** demonstrated high specific uptake relative to background signal within kidney, confirmed by immunohistochemistry. These compounds may be useful for FGS in prostate, renal or other PSMA-expressing cancers.

## 1. Introduction

As surgeons use robot-assisted laparoscopy in more operations and for an increasing number of indications, they are less likely to palpate the tumor and surgical bed to determine the extent of their resection. Accordingly, they must increasingly rely on visual inspection, which real-time fluorescence imaging can enhance by helping to avoid leaving behind a positive margin [1]. This is particularly true in the case of cancers curable only through surgery, such as colorectal cancer [2]. Advances in optical imaging instrumentation and molecular imaging agents are promoting the increased use of intraoperative, near-infrared (NIR) fluorescence imaging to help improve outcomes [3,4]. Most clinical studies employing fluorescence-guided surgery (FGS) involve the use of non-targeted protoporphyrins, such as 5-aminolevulinic acid, or cyanine dyes, such as indocyanine green, with quantum yields suitable for imaging in vivo to depths of between 0.5 and 1 cm [5,6]. However, there are a variety of available tumor-targeting moieties to which one may attach NIR dyes to confer a measure of specificity, including small molecules, peptides, proteins, antibodies, aptamers and nanobodies, among others [2,7]. One may also confer specificity of optical imaging agents through pharmacokinetic optimization in a structure-inherent targeting strategy, as recently shown for a small series of squaraine dyes [8]. The goal in designing such agents is to have one that will enable high tumor-to-background ratios at a convenient time after administration, usually within 12–24 h, for sensitive, real-time FGS.

Others and we have developed targeted NIR agents to enable specific evaluation of nerves, vascular structures within tumors, and tumor epithelium [9,10,11,12,13]. Regarding the last, the prostate-specific membrane antigen (PSMA) has received considerable attention as an optical imaging target because of its abundant expression on the surface of most prostate cancer cells and the synthetic accessibility of fluorescent conjugates of urea-based PSMA-binding affinity agents [13,14,15,16,17,18]. A PSMA-targeted NIR agent could be useful toward the end of a prostatectomy to help ensure a negative surgical margin. PSMA is also expressed in the neovascular endothelium of most solid tumors, such as lung, colon, pancreatic, renal carcinoma, and melanoma, but not in normal vasculature [19,20], so it might be a useful target in surgical guidance during the resection of non-prostate tumors as well.

We have previously tested our initial PSMA-targeted NIR agent, YC-27, which bears the IRDye 800CW fluorophore, in xenograft-bearing mice and showed that it was useful in preventing recurrence in the surgical margin [21]. We have also coupled it with concurrent NIR laser excitation and ultrasound to delineate tumors by photoacoustic imaging [22]. However, YC-27 may not represent an optimized structure with respect to pharmacokinetics or properties of the fluorophore. Because of its commercial availability and putatively higher fluorescence intensity and photostability [23], we switched to the DyLight800 fluorophore and altered the structure of the linker between the fluorophore and the PSMA-targeting moiety in a series of compounds to optimize performance, i.e., tumor visualization in vivo at 24 h post-injection. Our ultimate goal is to have an imaging agent with an optimized fluorophore and pharmacokinetics for real-time surgical guidance in a field that contains PSMA-expressing tissues.

## 2. Materials and Methods

### 2.1. Chemistry

General Methods. All chemicals and solvents were purchased from either Sigma-Aldrich (Milwaukee, WI) or Fisher Scientific (Pittsburgh, PA, USA). Boc-15-amino-4,7,10,13-tetraoxapentadecanoic acid and H-Lys(Boc)-Ot-Bu.HCl were purchased from Chem-Impex International (Wood Dale, IL, USA). *t*-Boc-*N*-amido-PEG_8_-acid was purchased from BroadPharm, Inc. (San Diego, CA, USA). The *N*-hydroxysuccinimide (NHS) ester of DyLight800 was purchased from Thermo Fisher Scientific (Rockford, IL, USA). Care was taken to limit the exposure of DyLight800-NHS and DyLight800-urea conjugates **1**–**10** from light. ^1^H NMR spectra were recorded on a Bruker Ultrashield 500 MHz spectrometer. ESI mass spectra were obtained on a Bruker Esquire 3000 plus system. (Billerica, MA, USA). High-performance liquid chromatography (HPLC) purifications were performed on a Varian Prostar System (Varian Medical Systems, Palo Alto, CA, USA).

DyLight800-**1**: To the trifluoroacetate salt of (*S*)-5-carboxy-5-(3-((*S*)-1,3-dicarboxypropyl)ureido)pentan-1-amine, **1** [24] (0.5 mg, 1.2 μmol) in DMSO (0.1 mL), *N*,*N*-diisopropylethylamine (0.010 mL, 57.4 μmol) was added, followed by the NHS ester of DyLight800 (0.3 mg, 0.29 μmol). After 1 h at room temperature, the stirred reaction mixture was purified by HPLC (column, Phenomenex Luna C18, 10 μ, 250 × 4.6 mm; mobile phase, A = 0.1% TFA in H_2_O, B = 0.1% TFA in CH_3_CN; linear gradient, 0 min = 5% B, 5 min = 5% B, 45 min = 100% B; flow rate, 1 mL/min) to afford 0.3 mg (87%) of DyLight800-**1**. ESI-Mass calculated for C_57_H_69_N_5_O_18_S_3_^2−^ [M-2H]^2−^ 603.7 found 603.6.

DyLight800-**2**: To a solution of (*S*)-*N*-(4-bromobenzyl)-5-carboxy-5-(3-((*S*)-1,3-dicarboxypropyl)ureido)pentan-1-amine **2** *(20)* (0.5 mg, 1.0 μmol) in DMSO (0.1 mL), *N*,*N*-diisopropylethylamine (0.005 mL, 28.7 μmol) was added, followed by the NHS ester of DyLight800 (0.3 mg, 0.29 μmol). After stirring overnight at room temperature, the reaction mixture was purified by HPLC (column, Phenomenex Luna C18, 10 μ, 250 × 4.6 mm; mobile phase, H_2_O/CH_3_CN/TFA = 67/33/0.1; flow rate, 1 mL/min) to afford 0.2 mg (51%) of DyLight800-**2**. ESI-Mass calculated for C_64_H_74_BrN_5_O_18_S_3_^2−^ [M-2H]^2−^ 687.7 found 687.5.

DyLight800-**3**: To the trifluoroacetate salt of (3*S*,7*S*,22*S*)-1,3,7,22-tetracarboxy-5,13,20-trioxo-4,6,12,21-tetraazahexacosan-26-amine **3** (*9*), (0.5 mg, 0.7 μmol) in DMSO (0.1 mL), *N,N*-diisopropylethylamine (0.010 mL, 57.4 μmol) was added, followed by the NHS ester of DyLight800 (0.3 mg, 0.29 μmol). After 1 h at room temperature, the stirred reaction mixture was purified by HPLC (column, Phenomenex Luna C18, 10 μ, 250 × 4.6 mm; mobile phase, A = 0.1% TFA in H_2_O, B = 0.1% TFA in CH_3_CN; linear gradient, 0 min = 5% B, 5 min = 5% B, 45 min = 100% B; flow rate,1 mL/min) to afford 0.3 mg (70%) of DyLight800-**3**. ESI-Mass calculated for C_71_H_94_N_7_O_22_S_3_^−^ [M-H]^−^ 1492.6 found 1492.4.

(3*S*,7*S*)-12-(4-Bromobenzyl)-1,3,7-tricarboxy-5,13,20-trioxo-4,6,12,21-tetraazahexacosan-26-aminium trifluoroacetate, **4**. To a solution of (*S*)-*N*-(4-bromobenzyl)-6-(tert-butoxy)-5-(3-((*S*)-1,5-di-tert-butoxy-1,5-dioxopentan-2-yl)ureido)-6-oxohexan-1-amine, **12** [25] (0.066 g, 0.1 mmol) in CH_2_Cl_2_ (4 mL), triethylamine (0.05 mL, 0.36 mmol) was added, followed by (*S*)-2,5-dioxopyrrolidin-1-yl 8-((1-(*tert*-butoxy)-6-((*tert*-butoxycarbonyl)amino)-1-oxohexan-2-yl)amino)-8-oxooctanoate, **11** (0.060 g, 0.11 mmol). After stirring overnight at room temperature, the solvent was evaporated and a solution of TFA/H_2_O (95:5, 0.3 mL) was added. The mixture was kept at room temperature for 1 h, then purified by HPLC (column, Phenomenex Luna C18, 10 μ, 250 × 10 mm; mobile phase, A = 0.1% TFA in H_2_O, B = 0.1% TFA in CH_3_CN; linear gradient, 0 min = 5% B, 5 min = 5% B, 25 min = 100% B; flow rate, 4 mL/min) to afford 0.048 g (55%) of **4**. ^1^H NMR (500 MHz, D_2_O, compound exists as a mixture of rotamers) δ 7.40–7.45 (m, 2H), 7.04 (m, 2H), 4.44–4.51 (m, 2H), 4.19–4.25 (m, 2H), 4.08 (m, 1H), 3.26 (m, 2H), 2.91 (m, 2H), 2.38–2.42 (m, 3H), 2.17–2.25 (m, 3H), 2.08–2.10 (m, 1H), 1.81–1.89 (m, 2H), 1.37–1.68 (m, 13H), 1.25 (m,4H). 1.14 (m, 2H). ESI-Mass calculated for C_33_H_51_BrN_5_O_11_^+^ [M + H]^+^ 772.3 found 772.2.

DyLight800-**4**: To a solution of **4** (0.5 mg, 0.57 μmol) in DMSO (0.1 mL), *N,N*-diisopropylethylamine (0.010 mL, 57.4 μmol) was added, followed by the NHS ester of DyLight800 (0.5 mg, 0.48 μmol). After stirring for 1 h at room temperature, the reaction mixture was purified by HPLC (column, Phenomenex Luna C18, 10 μ, 250 × 4.6 mm; mobile phase, A = 0.1% TFA in H_2_O, B = 0.1% TFA in CH_3_CN; linear gradient, 0 min = 5% B, 5 min = 5% B, 45 min = 100% B; flow rate, 1 mL/min) to afford 0.5 mg (63%) of DyLight800-**4**. ESI-Mass calculated for C_78_H_98_BrN_7_O_22_S_3_^2−^ [M-2H]^2−^ 829.9 found 830.5.

(3*S*,7*S*)-1,3,7-Tricarboxy-5,13,20-trioxo-4,6,12,21-tetraazahexacosan-26-aminium trifluoroacetate ***5***: To the formate of (*S*)-6-(*tert*-butoxy)-5-(3-((*S*)-1,5-di-*tert*-butoxy-1,5-dioxopentan-2-yl)ureido)-6-oxohexan-1-amine, **15** (0.027 g, 0.05 mmol) [24] in CH_2_Cl_2_ (2 mL), triethylamine (0.02 mL, 0.14 mmol) was added, followed by 2,5-dioxopyrrolidin-1-yl 8-((5-((*tert*-butoxycarbonyl)amino)pentyl)amino)-8-oxooctanoate,**13** (0.023 g, 0.05 mmol). After stirring at room temperature for 2 h, the solvent was evaporated and the residue was purified by a silica gel column (5% MeOH in CH_2_Cl_2_) to give the protected intermediate (0.037 g, 0.044mmol) 88%). ESI-Mass calculated for C_42_H_78_N_5_O_11_ [M + H]^+^ 828.6 found 828.6. To this, a solution of TFA/H_2_O (95:5, 0.3 mL) was added. The mixture was stirred at ambient temperature for 2 h, then purified on a Sep-Pak Vac 12 cc syringe column (Waters) using a gradient of water to acetonitrile/water 5:5 (*v*:*v*) to give compound **5** (0.023 g, 78%). ^1^H NMR (500 MHz, D_2_O/CD_3_CN = 2:1) δ 3.97–4.02 (m, 2H), 3.05 (m, 4H), 2.83–2.86 (m, 2H), 2.31 (m, 2H), 2.09 (m, 4H), 1.79 (m, 1H), 1.66 (m, 1H), 1.40–1.56 (m, 12H), 1.19–1.28 (m, 8H). ESI-Mass calculated for C_25_H_46_N_5_O_9_^+^ [M + H]^+^ 560.3 found 560.3.

DyLight800-**5**: To a solution of compound 5 (0.5 mg, 0.74 μmol) in DMSO (0.1 mL), *N*,*N*-diisopropylethylamine (0.010 mL, 57.4 μmol) was added, followed by the NHS ester of DyLight800 (0.3 mg, 0.29 μmol). After stirring 2 h at room temperature, the reaction mixture was purified by HPLC (column, Phenomenex Luna C18, 10 μ, 250 × 4.6 mm; mobile phase, A = 0.1% TFA in H_2_O, B = 0.1% TFA in CH_3_CN; linear gradient, 0 min = 5% B, 5 min = 5% B, 25 min = 100% B; flow rate, 1 mL/min) to afford 0.3 mg (72%) of DyLight800-**5**. ESI-Mass calculated for C_70_H_93_N_7_O_20_S_3_^2−^ [M-2H]^2−^, 723.8, found 723.6.

(3*S*,7*S*)-12-(4-Bromobenzyl)-1,3,7-tricarboxy-5,13,20-trioxo-4,6,12,21-tetraazahexacosan-26-aminium trifluoroacetate, **6**: To a solution of compound **12** (0.016 g, 0.024 mmol) in CH_2_Cl_2_ (2 mL), triethylamine (0.01 mL, 0.072 mmol) was added, followed by **13** (0.011 g, 0.024 mmol). After stirring at room temperature for 2 h, the solvent was evaporated and the residual was purified by HPLC (column, Phenomenex Luna C18, 10 μ, 250 × 10 mm; mobile phase, A = 0.1% TFA in H_2_O, B = 0.1% TFA in CH_3_CN; linear gradient, 0 min = 40% B, 5 min = 40% B, 25 min = 100% B; flow rate, 4 mL/min) to give 0.013 g (54%) of the protected intermediate. ESI-Mass calculated for C_49_H_83_BrN_5_O_11_ [M + H]^+^ 996.5 found 996.4. To the protected intermediate, a solution of TFA/H_2_O (95:5, 0.3 mL) was added. The mixture was kept at ambient temperature for 2 h, then purified on a Sep-Pak Vac 12 cc syringe column (Waters) using a gradient of water to acetonitrile/water of 5:5 (*v*:*v*) to give compound **6** (0.008 g, 73%). ^1^H NMR (500 MHz, D_2_O/CD_3_CN = 1:1, compound exists as a mixture of rotamers) δ 7.43–7.49 (m, 2H), 7.08 (m, 2H), 4.43–4.49 (m, 2H), 4.02–4.08 (m, 2H), 3.18–3.26 (m, 2H), 3.04–3.08 (m, 2H), 2.82–2.85 (m, 2H), 2.23–2.36 (m, 4H), 2.08–2.11 (m, 2H), 1.79–1.82 (m, 1H), 1.66 (m, 1H),1.49–1.58 (m, 12H), 1.13–1.26 (m, 8H). ESI-Mass calculated for C_32_H_51_BrN_5_O_9_^+^ [M + H]^+^ 728.3 found 728.2.

DyLight800-**6**: To a solution of compound 6 (0.5 mg, 0.59 µmol) in DMSO (0.1 mL), *N,N*-diisopropylethylamine (0.010 mL, 57.4 μmol) was added, followed by the NHS ester of DyLight800 (0.3 mg, 0.29 μmol). After stirring 2 h at room temperature, the reaction mixture was purified by HPLC (column, Phenomenex Luna C18, 10 μ, 250 × 4.6 mm; mobile phase, A = 0.1% TFA in H_2_O, B = 0.1% TFA in CH_3_CN; linear gradient, 0 min = 5% B, 5 min = 5% B, 25 min = 100% B; flow rate, 1 mL/min) to afford 0.4 mg (86%) of DyLight800-**6**. ESI-Mass calculated for C_77_H_98_BrN_7_O_20_S_3_^2−^ [M-2H]^2−^, 807.8, found 808.6.

(21*S*,25*S*)-21,25,27-Tricarboxy-15,23-dioxo-3,6,9,12-tetraoxa-16,22,24-triazaheptacosan-1-aminium trifluoroacetate, **7**: To a solution of 15 (0.053 g, 0.1 mmol) in CH_2_Cl_2_ (2 mL), triethylamine (0.027 mL, 0.2 mmol) was added, followed by 2,5-dioxopyrrolidin-1-yl 2,2-dimethyl-4-oxo-3,8,11,14,17-pentaoxa-5-azaicosan-20-oate, **17** (0.046 g, 0.1 mmol). After stirring at room temperature for 2 h, the solvent was evaporated and the residue was purified by a silica gel column (5% MeOH in CH_2_Cl_2_) to give the protected intermediate (0.051 g, 61%). ESI-Mass calculated for C_40_H_74_N_4_O_14_Na [M + Na]^+^ 857.5 found 857.5. To the protected intermediate, a solution of TFA/H_2_O (95:5, 0.2 mL) was added. The mixture was stirred at ambient temperature for 2 h, then purified using a Sep-Pak Vac 12 cc syringe column (Waters) using a gradient of 100% water to acetonitrile/water 3:7 (*v*:*v*). Product fractions were collected and lyophilized to give compound **7** (0.032 g, 79%). ^1^H NMR (500 MHz, D_2_O) δ 4.20 (m, 1H), 4.12 (m, 1H), 3.76 (m, 4H), 3.65–3.70 (m, 12H), 3.17–3.20 (m, 4H), 2.46–2.50 (m, 4H), 2.12–2.15 (m, 1H), 1.92–1.98 (m, 1H), 1.78–1.83 (m, 1H), 1.64–1.72 (m, 1H), 1.49–1.55 (m, 2H), 1.37–1.41 (m, 2H). ESI-Mass calculated for C_23_H_43_N_4_O_12_^+^ [M + H]^+^ 567.3 found 567.2.

DyLight800-**7**: To a solution of compound **7** (0.3 mg, 0.45 μmol) in DMSO (0.1 mL), *N*,*N*-diisopropylethylamine (0.010 mL, 57.4 μmol) was added, followed by the NHS ester of DyLight800 (0.3 mg, 0.29 μmol). After stirring for 2 h at room temperature, the reaction mixture was purified by HPLC (column, Phenomenex Luna C18, 10 μ, 250 × 4.6 mm; mobile phase, A = 0.1% TFA in H_2_O, B = 0.1% TFA in CH_3_CN; gradient, 0 min = 5% B, 5 min = 5% B, 45 min = 100% B; flow rate, 1 mL/min) to afford 0.3 mg (72%) of DyLight800-**7**. ESI-Mass calculated for C_68_H_90_N_6_O_23_S_3_^2−^ [M-2H]^2−^ 727.3 found 727.1.

(21*S*,25*S*)-16-(4-Bromobenzyl)-21,25,27-tricarboxy-15,23-dioxo-3,6,9,12-tetraoxa-16,22,24-triazaheptacosan-1-aminium trifluoroacetate*,* **8**: To a solution of compound **12** (0.066 g, 0.1 mmol) in CH_2_Cl_2_ (2 mL), triethylamine (0.027 mL, 0.2 mmol) was added, followed by 2,5-dioxopyrrolidin-1-yl 2,2-dimethyl-4-oxo-3,8,11,14,17-pentaoxa-5-azaicosan-20-oate, **17** (0.046 g, 0.1 mmol). After stirring for 2 h at room temperature, the solvent was evaporated, and the residue was purified by a silica gel column (5% MeOH in CH_2_Cl_2_) to give the protected intermediate (0.082 g, 81%) ESI-Mass calculated for C_47_H_79_BrN_4_O_14_Na^+^ [M + Na]^+^ 1025.5 found 1025.3. To the protected intermediate, a solution of TFA/H_2_O (95:5, 0.2 mL) was added. The mixture was kept at an ambient temperature for 2 h, and the solvent was evaporated and the residue purified on a Sep-Pak Vac 12 cc syringe column (Waters) using a gradient of 100% water to acetonitrile/water 1:1 (*v*:*v*). The fractions were collected and lyophilized to give compound **8** (0.056 g, 67%). ^1^H NMR (500 MHz, D_2_O, compound exists as a mixture of rotamers) δ 7.49–7.55 (m, 2H), 7.12–7.15 (m, 2H), 4.52–4.61 (m, 2H), 4.10 (m, 1H), 4.03 (m, 1H), 3.68–3.80 (m, 4H), 3.54–3.64 (m, 12H), 3.32–3.38 (m, 2H), 3.13–3.15 (m, 2H), 2.62–2.76 (m, 2H), 2.37–2.40 (m, 2H), 2.04–2.08 (m, 1H), 1.86–1.89 (m, 1H), 1.67–1.73 (m, 1H), 1.51–1.61 (m, 3H), 1.26–1.30 (m, 2H). ESI-Mass calculated for C_30_H_48_BrN_4_O_12_^+^ [M + H]^+^ 735.2 found 735.2.

DyLight800-**8**: To a solution of compound **8** (0.5 mg, 0.60 μmol) in DMSO (0.1 mL), *N*,*N*-diisopropylethylamine (0.010 mL, 57.4 μmol) was added, followed by the NHS ester of DyLight800 (0.3 mg, 0.29 μmol). After stirring for 2 h at room temperature, the reaction mixture was purified by HPLC (column, Phenomenex Luna C18, 10 μ, 250 × 4.6 mm; mobile phase, A = 0.1% TFA in H_2_O, B = 0.1% TFA in CH_3_CN; gradient, 0 min = 5% B, 5 min = 5% B, 45 min = 100% B; flow rate, 1 mL/min) to afford 0.3 mg (65%) of DyLight800-**8**. ESI-Mass calculated for C_75_H_95_BrN_6_O_23_S_3_^2−^ [M-2H]^2−^ 811.2 found 811.0.

2,5-Dioxopyrrolidin-1-yl 2,2-dimethyl-4-oxo-3,8,11,14,17,20,23,26,29-nonaoxa-5-azadotriacontan-32-oate, **20**: To a solution of *t*-Boc-*N*-amido-PEG_8_-acid (0.25 g, 0.46 mmol) and *N*-hydroxysuccinimide (0.053 g, 0.046 mmol) in CH_2_Cl_2_ (4 mL), dicyclohexylcarbodiimide (0.1 g, 0.048 mmol) was added. After stirring at room temperature overnight, the mixture was filtered, and the filtrate was evaporated to give compound **20**. ESI-Mass calculated for C_28_H_50_N_2_O_14_Na [M + Na]^+^ 661.3 found 661.3.

(33*S*,37*S*)-33,37,39-Tricarboxy-27,35-dioxo-3,6,9,12,15,18,21,24-octaoxa-28,34,36-triazanonatriacontan-1-aminium trifluoroacetate **9**: To a solution of the formate salt of **15** (0.053 g, 0.1 mmol) in CH_2_Cl_2_ (2 mL), triethylamine (0.027 mL, 0.2 mmol) was added, followed by 2,5-dioxopyrrolidin-1-yl 2,2-dimethyl-4-oxo-3,8,11,14,17,20,23,26,29-nonaoxa-5-azadotriacontan-32-oate, **20** (0.070 g, 0.11 mmol). After 2 h of stirring at room temperature, the solvent was evaporated, and the residual was purified by a silica gel column (5% MeOH in CH_2_Cl_2_) to give the protected intermediate (0.076 g, 76%). A solution of TFA/H_2_O (95:5, 0.2 mL) was added to the protected intermediate (0.060 g, 0.059 mmol). The mixture was kept at an ambient temperature for 2 h, then purified by Sep-Pak Vac 12 cc (Waters) using a gradient of water to acetonitrile/water of 3:7 (*v*:*v*). The fractions were collected and lyophilized to give compound **9** (0.036 g, 72%). ^1^H NMR (500 MHz, D_2_O) δ 4.12 (m, 1H), 4.07 (m, 1H), 3.76 (m, 4H), 3.65–3.70 (m, 28H), 3.17–3.20 (m, 4H), 2.48–2.50 (m, 2H), 2.41–2.44 (m, 2H), 2.06–2.10 (m, 1H), 1.88–1.92 (m, 1H), 1.76–1.79 (m, 1H), 1.65–1.69 (m, 1H), 1.49–1.53 (m, 2H), 1.35–1.38 (m, 2H). ESI-Mass calculated for C_31_H_59_N_4_O_16_^+^ [M + H]^+^ 743.4 found 743.3.

DyLight800-**9**: To a solution of compound **9** (0.5 mg, 0.60 μmol) in DMSO (0.1 mL), *N*,*N*-diisopropylethylamine (0.010 mL, 57.4 μmol) was added, followed by the NHS ester of DyLight800 (0.3 mg, 0.29 μmol). After stirring for 2 h at room temperature, the reaction mixture was purified by HPLC (column, Phenomenex Luna C18, 10 μ, 250 × 4.6 mm; mobile phase, A = 0.1% TFA in H_2_O, B = 0.1% TFA in CH_3_CN; gradient, 0 min = 5% B, 5 min = 5% B, 45 min = 100% B; flow rate, 1 mL/min) to afford 0.4 mg (86%) of DyLight800-**9**. ESI-Mass calculated for C_76_H_106_N_6_O_27_S_3_^2−^ [M-2H]^2−^ 815.3 found 815.1.

(33S,37*S*)-28-(4-Bromobenzyl)-33,37,39-tricarboxy-27,35-dioxo-3,6,9,12,15,18,21,24-octaoxa-28,34,36-triazanonatriacontan-1-aminium trifluoroacetate **10**: To a solution of compound 12 (0.066 g, 0.1 mmol) in CH_2_Cl_2_ (3 mL), triethylamine (0.027 mL, 0.2 mmol) was added, followed by 2,5-dioxopyrrolidin-1-yl 2,2-dimethyl-4-oxo-3,8,11,14,17,20,23,26,29-nonaoxa-5-azadotriacontan-32-oate, **20**, (0.070 g, 0.11 mmol). After stirring overnight at room temperature, the solvent was evaporated, and the residue was purified on a silica gel column (5% MeOH in CH_2_Cl_2_) to give the protected intermediate (0.094 g, 79%). To the protected intermediate (0.07 g, 0.059 mmol), a solution of TFA/H_2_O (95:5, 0.2 mL) was added. The mixture was kept at ambient temperature for 2 h, then purified by HPLC (column: Phenomenex Luna C18, 10 μ, 250 × 10 mm; mobile phase, A = 100% water + 0.1%TFA, B = 100% acetonitrile + 0.1% TFA, Linear Gradient: 0 min 100%A, 25 min 100%B; flow rate, 4 mL/min) to give compound 10 (0.031g, 52%). ^1^H NMR (500 MHz, D_2_O, compound exists as a mixture of rotamers) δ 7.49–7.53 (m, 2H), 7.12–7.16 (m, 2H), 4.52–4.61 (m, 2H), 4.21 (m, 1H), 4.10 (m, 1H), 3.71–3.80 (m, 4H), 3.54–3.66 (m, 28H), 3.32–3.38 (m, 2H), 3.15–3.16 (m, 2H), 2.63–2.78 (m, 2H), 2.44–2.47 (m, 2H), 2.09–2.15 (m, 1H), 1.88–1.96 (m, 1H), 1.71–1.77 (m, 1H), 1.50–1.64 (m, 3H), 1.28–1.30 (m, 2H). ESI-Mass calculated for C_38_H_64_BrN_4_O_16_^+^ [M + H]^+^ 912.9 found 913.3.

DyLight800-**10**: To a solution of compound **10** (0.5 mg, 0.50 μmol) in DMSO (0.1 mL), *N*,*N*-diisopropylethylamine (0.010 mL, 57.4 μmol), followed by the NHS ester of DyLight800 (0.3 mg, 0.29 μmol) was added. After stirring for 2 h at room temperature, the reaction mixture was purified by HPLC (column, Phenomenex Luna C18, 10 μ, 250 × 4.6 mm; mobile phase, A = 0.1% TFA in H_2_O, B = 0.1% TFA in CH_3_CN; gradient, 0 min = 5% B, 5 min = 5% B, 45 min = 100% B; flow rate, 1 mL/min) to afford 0.3 mg (58%) of DyLight800-**10**. ESI-Mass calculated for C_83_H_111_BrN_6_O_27_S_3_^2−^ [M-2H]^2−^ 899.3 found 899.1.

Synthetic details for the linkers used in compounds **4**–**10** are presented in the Supplemental Materials.

### 2.2. NAALADase Assay

Cell lysates of LNCaP cell extracts were incubated with PSMA-targeted imaging agents (0.01 nM–100 μM) in the presence of 4 μM NAAG at 37 °C for 2 h. The amount of released glutamate from NAAG was measured by incubating with a working solution of the Amplex Red glutamic acid kit (Molecular Probes Inc., Eugene, OR, USA) at 37 °C for 60 min. Fluorescence was determined by reading with the Cytation 5 Cell Imaging Multi-Mode Reader (BioTek, Winooski, VT, USA) with excitation at 545 nm and emission at 590 nm [26]. Inhibition curves were determined using semi-log plots, and IC_50_ values were determined as the concentration at which enzymatic activity was inhibited by 50%. Assays were performed in triplicate, with the entire inhibition study being repeated at least once to confirm the affinity and mode of inhibition. Enzyme inhibitory constants (*K*_i_ values) were generated using the Cheng–Prusoff conversion [27]. Data analysis was performed using GraphPad Prism version 9 for Windows (GraphPad Software, San Diego, CA, USA).

### 2.3. Cell Lines and Tumor Models

PSMA^+^ PC3 PIP and PSMA^−^ PC3 flu cell lines were originally obtained from Dr. Warren Heston (Cleveland Clinic). Cells were grown to 80–90% confluence in a single passage before trypsinization and formulation in Hank’s balanced salt solution (HBSS, Sigma, St. Louis, MO, USA) for implantation into mice. PC3-ML-PSMA [28] cells maintained in RPMI1640 supplemented with 10% FBS and 1× Pen/Strep. Animal studies were carried out in compliance with guidelines related to the conduct of animal experiments of the Johns Hopkins Animal Care and Use Committee. For optical imaging studies and ex vivo biodistribution, male NOD-SCID mice (Johns Hopkins University, in-house colony) were implanted subcutaneously with 1 × 10^6^ PSMA^+^ PC3 PIP and PSMA^−^ PC3 flu cells in opposite flanks. Mice were imaged when the tumor xenografts reached 3–5 mm in diameter. For the metastatic model of a PSMA-expressing prostate tumor, 1 × 10^6^ PC3-ML-PSMA cells were injected intravenously into 4 to 6-week-old NSG (NOD/Shi-scid/IL-2Rγnull) mice (Animal Resources Core, Johns Hopkins). Four weeks post-injection of the cells, animals were used for imaging.

### 2.4. In Vivo Imaging and Ex Vivo Biodistribution

After image acquisition at baseline (pre-injection), each mouse was injected intravenously with 1 nmol of DyLight800-urea conjugate (DyLight800-**1** to DyLight800-**10**, **3**–**4** mice per compound), and images were acquired at 1 h, 2 h, 4 h and 24 h time points using a Pearl Impulse Imager (LI-COR Biosciences, Cambridge, UK). Following the 24 h image, each mouse was sacrificed by cervical dislocation, and tumor, muscle, liver, spleen, kidneys and stomach were collected and assembled on a petri dish for image acquisition. All images were scaled to the same intensity for direct comparison.

### 2.5. Immunohistochemistry (IHC)

The kidney and liver were fixed in 10% formalin for 24 h and embedded in paraffin. Sectioning and H&E staining were performed by Johns Hopkins Oncology Tissue Services. For PSMA IHC, slides were stained with anti-PSMA antibody (Cat# SIR089, Dako, Santa Clara, CA, USA) with 1:2 dilution in the presence of background reducing components (Cat# S3022, Dako). Slides were then stained with anti-mouse HRP linked antibody from EnVision plus system-HRP (DAB) (Cat# K4006, Dako). Processed slides were scanned by Johns Hopkins Oncology Tissue Services, and the images were analyzed using Aperio Image Scope software (Leica Biosystems Inc, Buffalo Grove, IL, USA).

### 2.6. Statistical Considerations

An unpaired *t*-test was performed to determine whether there were differences in PSMA^+^ PC3 PIP tumor uptake and the kidney and corresponding PSMA^−^ PC3 flu tumor for DyLight800-**1**–**10**. Statistical significance was determined to be present at *p* < 0.01. Calculations were performed using GraphPad Prism 9 (San Diego, CA, USA).

## 3. Results

### 3.1. Chemical Synthesis

**Scheme 1:**
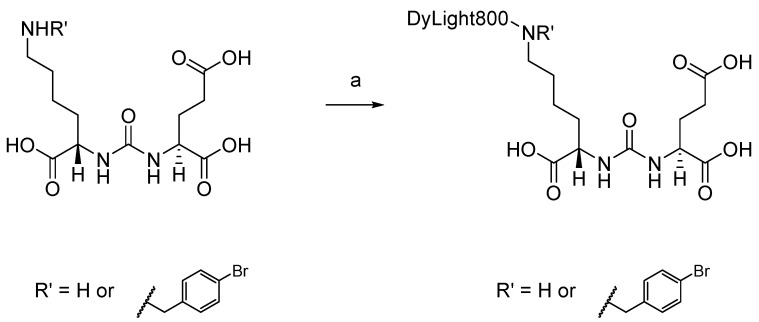
General syntheses of DyLight800-**1** (R’ = H) and -**2** (no linker, R’ = *para*-BrBn).

**Scheme 2:**
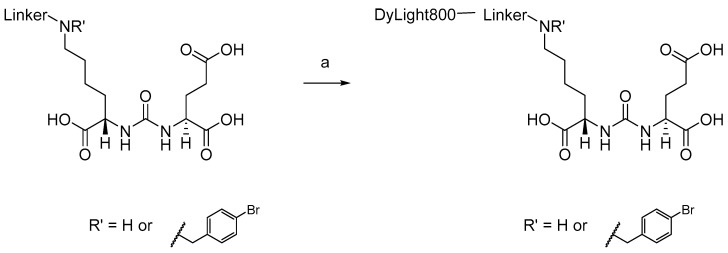
General syntheses of DyLight800-**3** to -**10**. (See Figure 1 for R’ and linkers).

General syntheses of DyLight800-**1** and -**2** (no linker) are presented in Scheme 1, while general syntheses of DyLight-**3** through -**10** (with linker) are shown in Scheme 2.

**Figure 1:**
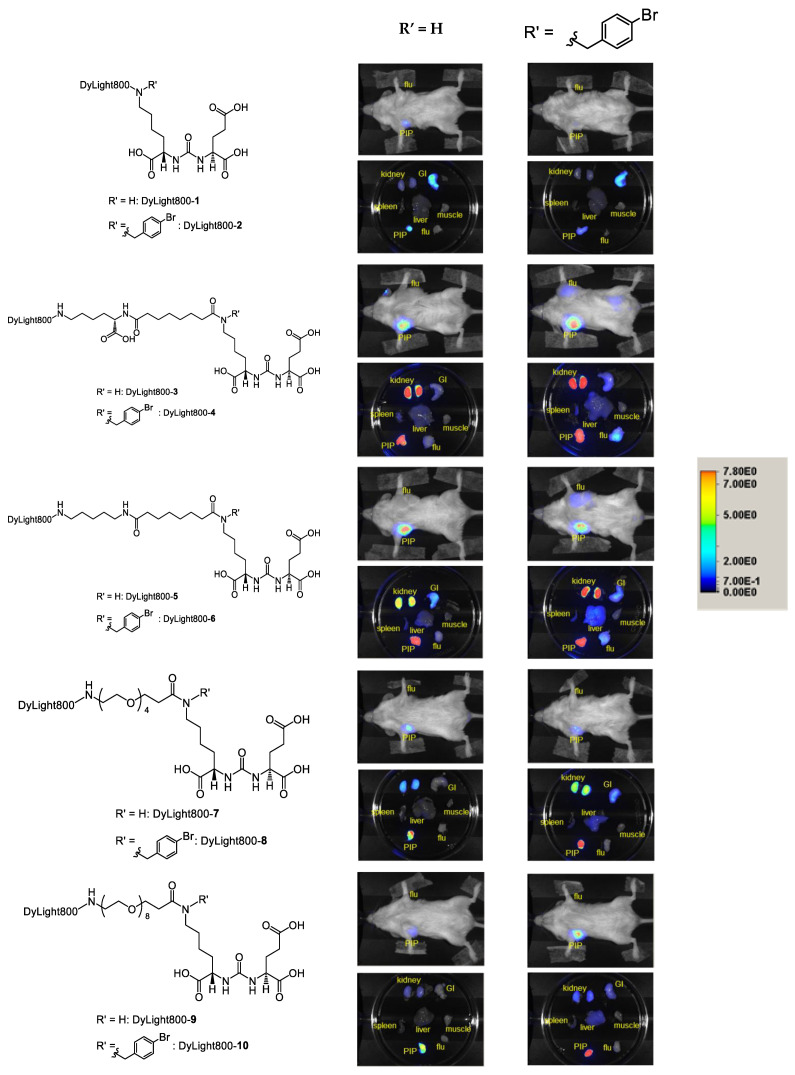
Whole body and excised organ imaging of mice with PSMA^+^ PC3 PIP and PSMA- PC3 flu tumors at 24 h post-injection of 1 nmol of DyLight800-urea conjugates.

Commercially available amine-reactive active NHS ester of DyLight800 with principal excitation/emission wavelength at 777/794 nm was conjugated with amines **1**–**10** to produce the dye-PSMA inhibitors shown in Figure 1. The conjugation reactions were readily completed at room temperature. The chemical yields for the conjugates ranged from 51% to 86%.

### 3.2. In Vitro Inhibition Assay

The *K*_i_ values were determined using a modification of the Amplex Red glutamic acid assay [26] and are presented in Table 1. The *K*_i_ values range from 0.10 to 2.19 nM, which is similar to other compounds of this class [29].

### 3.3. Imaging

**Figure 2:**
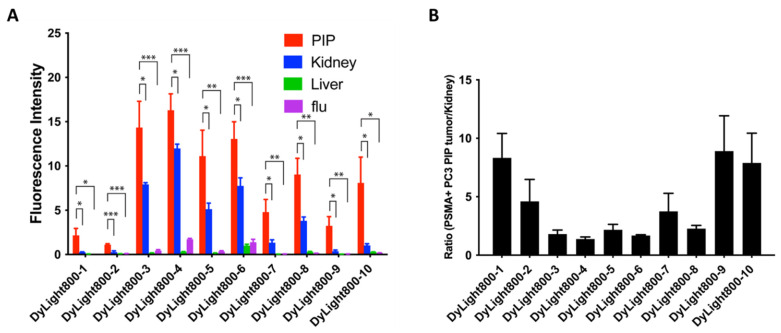
(**A**) Biodistribution data at 24 h postinjection from regions of interest (ROI) drawn over organs displayed in ex vivo images. Three to four animals were imaged per agent. Comparisons were made between the tumor and kidney, as well as between PSMA+ PC3 PIP tumor and kidney and between PSMA^+^ PC3 PIP and PSMA^−^ PC3 flu tumor. Values are represented as mean ± standard error of the mean (SEM). PIP = PSMA^+^ PC3 PIP tumor; flu = PSMA^−^ PC3 flu tumor. * = *p* < 0.01; ** = *p* < 0.001; *** = *p* < 0.0001. (**B**) Ratio of uptake in PSMA^+^ PC3 PIP tumor to kidney based on the data in panel (**A**).

Figure 1 shows the imaging at 24 h post-injection of 1 nmol of compound DyLight800-**1** to DyLight800-**10** in mice with PSMA^+^ PC3 PIP and PSMA^-^ PC3 flu tumors. All ten compounds demonstrated robust PSMA^+^ PC3 PIP tumor uptake and little uptake in PSMA^-^ PC3 flu tumors, indicating target selectivity in vivo. Fluorescence intensities of PIP, flu, kidney and liver are shown in Figure 2A. We found significant differences in uptake of each agent between the PSMA-expressing tumors and kidney and between these tumors and the PSMA-negative tumors according to the levels of significance indicated.

**Figure 3:**
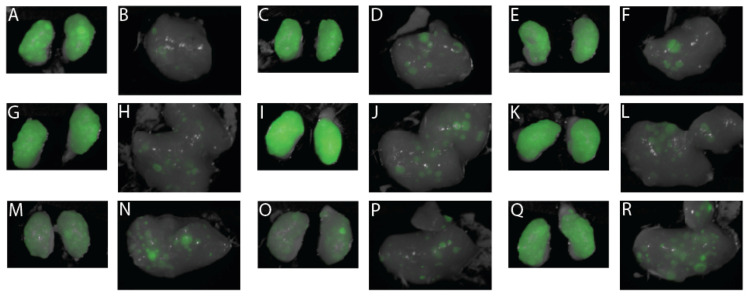
Selected agents successfully detected PSMA-expressing metastatic lesions in the kidney and liver. Ex vivo fluorescent images of kidney (**A**,**C**,**E**,**G**,**I**,**K**,**M**,**O**,**Q**) and liver (**B**,**D**,**F**,**H**,**J**,**L**,**N**,**P**,**R**) from mice injected with 1 nmole of compounds DyLight800-**4** (**A**–**F**), DyLight800-**6** (**G**–**L**), and DyLight800-**10** (**M**–**R**). Fluorescence intensity was optimized to maximize the contrast between tumor and kidney for each image.

**Figure 4:**
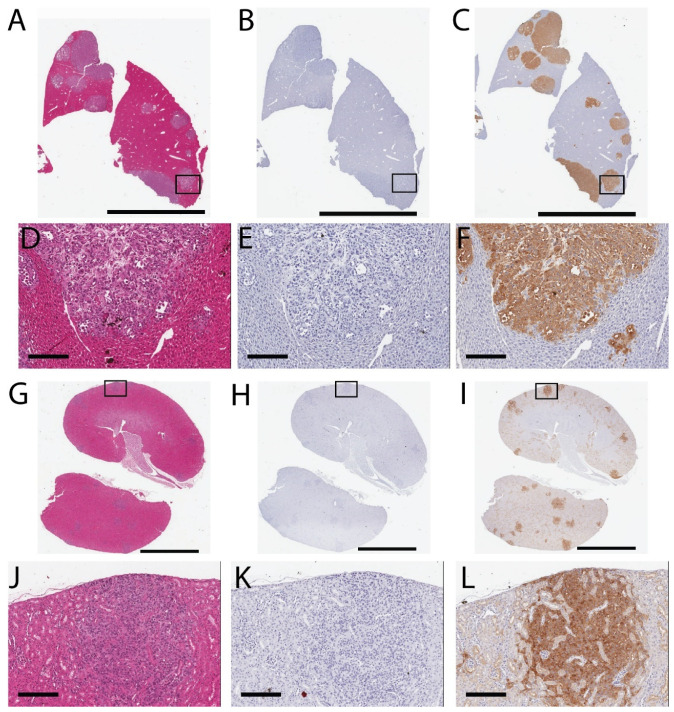
Immunohistochemistry confirmed the expression of PSMA in metastatic tumors and the cortex regions of mouse kidney. H&E staining (**A**,**D**,**G**,**J**), control staining without the 1° (anti-PSMA) Ab (**B**,**E**,**H**,**K**), and staining with anti-PSMA Ab (**C**,**F**,**I**,**L**). Scanned images of a whole section with 0.5× magnification (**A**–**C** and **G**–**I**, scale bar = 5 mm) and magnified images of boxed regions (**D**,**E**,**F**,**J**,**K**,**L**, scale bar = 200 μm) are shown.

As noted above, the key consideration for a suitable FGS agent is tumor/background at the time of surgery. The agent must also have high absolute tumor uptake to be detectable. DyLight800-**3**, DyLight800-**4**, DyLight800-**5** and DyLight800-**6** demonstrated the highest PSMA^+^ PIP tumor uptake. Comparing DyLight800-**7** and DyLight800-**9** with DyLight800-**8** and DyLight800-**10**, it was demonstrated that the *N*-bromobenzyl substituent significantly increased the PSMA^+^ PIP tumor uptake. DyLight800-**1** and DyLight800-**2**, which have no linker between the dye and Lys-Glu urea, had much lower PSMA^+^ PIP tumor uptake than other compounds with linkers, confirming the importance of the linker moiety for modifying pharmacokinetics. DyLight800-**7**, DyLight800-**8**, DyLight800-**9** and DyLight800-**10**, which have PEG_4_ or PEG_8_ linker had significantly less kidney uptake compared to DyLight800-**3**, DyLight800-**4**, DyLight800-**5** and DyLight800-**6**. While Dylight800-**1**, Dylight800-**9** and DyLight800-**10** showed the most favorable tumor/background, their absolute uptake levels were relatively low. Nevertheless, for renal surgery, one of them, particularly DyLight-**10**, may have the best properties (Figure 2B). DyLight800-**3** or DyLight800-**4** may be superior for prostate surgery, due to higher absolute uptake where renal uptake is of no consequence. Figure 3 shows the capacity for the dyes to identify lesions within metastatic foci of liver and kidney, while Figure 4 provides correlative histology from this experiment.

**Figure 5:**
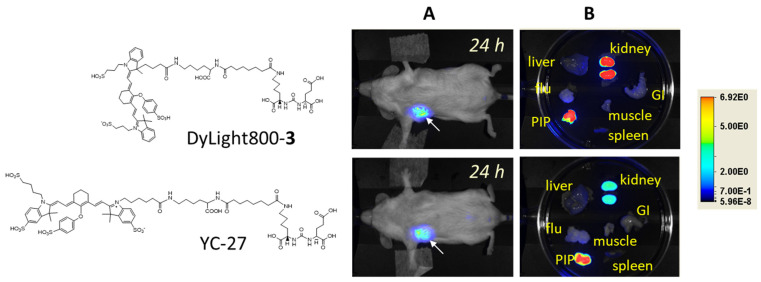
Head-to-head comparison of DyLight800-3 to YC-27. In each case, 1 nmol of agent was administered intravenously. Compounds provided comparable PSMA^+^ PC3 PIP tumor (white arrows in column **A**) uptake at 24 h post-injection. At 24 h, animals were sacrificed and organs were removed and imaged (column **B**). Images are scaled to the same maximum value.

We also compared the uptake of DyLight800-**3** to that of our previously published compound YC-27 (Figure 5) [13]. Qualitatively, the uptake in tumor of DyLight800-**3** is similar to or slightly higher than that of YC-27, while renal uptake is much lower for the latter. Note that these two compounds differ only in the fluorophore, with YC-27 bearing the IRDye 800CW dye.

## 4. Discussion

FGS is increasingly employed for a variety of conditions, particularly in surgical oncology. As of today, Clinical trials.gov lists 53 active or recent studies involving FGS, with likely even more ongoing [30]. As with most imaging modalities, in order to be successful, high sensitivity and specificity are required for the NIR agent and the detection system. Here we have focused on optimization of what we have shown is a highly specific fluorescent platform for targeting PSMA [13,14,15]. We have done so in two ways: first, by replacing our initial fluorophore with DyLight800, due to its putatively superior quantum yields and photostability [23] and its ready availability. Second, we attempted to enhance the pharmacokinetics by altering the linker between the fluorophore and the PSMA-targeting moiety. We did so by altering the linker length, its overall degree of hydrophilicity and by affixing a bromobenzyl group to the ε-amino group of lysine, to which we also attached the fluorophore. We have previously shown that the *N*-bromobenzyl group in that position is capable of mitigating off-target tissue uptake [31].

Although these agents are suggested for use in enhancing surgery, which means that in the context of prostate cancer, only local images in the tumor margin or of local-regional lymph nodes would be obtained, since PSMA is expressed in the neovasculature of many other tumors, it is important to minimize uptake in other organs, such as the kidney. For example, we have previously shown that we can target clear cell renal cell carcinoma (ccRCC) to good effect with a positron-emitting version of our PSMA-targeting ureas, DCFPyL (piflufolostat F 18) [32,33,34], and could potentially use an optimized NIR version for surgical guidance in partial nephrectomy, for which non-targeted carbocyanine dyes have been leveraged [11]. In this instance, DyLight800-**10** may be the best agent among those synthesized here, as there is little renal uptake yet sufficient brightness to enable clear visualization of metastatic foci (Figure 2). That compound arguably demonstrates the highest signal-to-noise ratio. For prostate surgery, DyLight800-**3** or DyLight800-**3**–**4**, which contain an acid group in the linker, may be superior, as renal uptake would be of no consequence in the surgical field. Nevertheless, that high renal uptake may be indicative of other non-target binding, which would have to be evaluated further. In all, it appears that the presence of the longer linker and enhanced hydrophilicity, such as the introduction of an acid group or a polyethylene glycol (PEG) chain in the linker, improve pharmacokinetics. The presence of the *N*-bromobenzyl group increased PSMA^+^ PIP tumor uptake. We hope that inclusion of the *N*-bromobenzyl substituent will also promote a decrease in salivary gland uptake, as it has for our corresponding radiotheranostics [31].

One strategy to improve the performance of PSMA-targeting agents for FGS is to use a fluorescent molecular rotor [35], which provides nearly instantaneous detection upon binding of PSMA. However, the clinical use and utility of such an agent may still be dictated by pharmacokinetics if administered intravenously. We must wait on the order of 12 to 24 h after administration for imaging in these preclinical models for clearance from non-target tissues. Others have developed NIR cancer detection agents that are merely sprayed onto the surgical site, further enhancing their convenience [36,37,38]. Whether those, or frankly any of these agents, actually improve outcomes measured by overall or progression-free survival remains to be determined in larger, prospective studies [5].

We undertook this study to optimize a PSMA-targeting NIR agent with respect to fluorophore and pharmacokinetics. Our qualitative, head-to-head comparison of DyLight800-**3** to YC-27 did not reveal a substantial difference in tumor uptake, although the latter had significantly lower renal uptake. The literature also provides little evidence for the superiority of DyLight800 over IRDye 800CW, and actually suggests the contrary in terms of quantum yield and brightness in certain in vitro assays [39,40]. Remaining, however, is the possibility that the issue of coupling a particular dye with a certain camera may further enhance sensitivity, while we used the Pearl Impulse Imager for our studies herein.

## 5. Conclusions

In conclusion, inclusion of an *N*-bromobenzyl substituent promoted increased PSMA^+^ PIP tumor uptake, while a hydrophilic linker, particularly with the inclusion of PEG, may decrease nonspecific binding, particularly to the kidney, in this new series of PSMA-targeted NIR agents. Such modifications, perhaps coupled with what could be a truly superior flurophore [40], may render this latest generation of NIR probes even more suitable for FGS in prostate cancer, ccRCC or other PSMA-expressing tumors than existing agents.

## Figures and Tables

**Table 1:** PSMA inhibitory activities.

Compound	*K*_i_ (nM)	95% Confidence Interval of *K*_i_
DyLight800-**1**	0.37	0.26–0.52
DyLight800-**2**	0.16	0.11–0.22
DyLight800-**3**	0.10	0.06–0.15
DyLight800-**4**	0.29	0.17–0.51
DyLight800-**5**	0.45	0.31–0.65
DyLight800-**6**	0.68	0.40–1.17
DyLight800-**7**	0.85	0.52–1.36
DyLight800-**8**	0.50	0.34–0.73
DyLight800-**9**	2.19	1.22–2.71
DyLight800-**10**	0.83	0.73–0.94

## Data Availability

Not applicable.

## Supplementary Materials

The following supporting information can be downloaded at: https://www.mdpi.com/article/10.3390/biom12030405/s1. Scheme S1: Reagent and conditions and corresponding synthetic detail.
