# Supplementary material for: A Series of PSMA-Targeted Near-Infrared Fluorescent Imaging Agents
Source: Biomolecules. 2022 Mar 5;12(3):405. doi: 10.3390/biom12030405 (PMC8946146; doi:10.3390/biom12030405)
Supplement: Supplementary file 1 [file biomolecules-12-00405-s001.zip › biomolecules-1571483-supplementary.pdf]

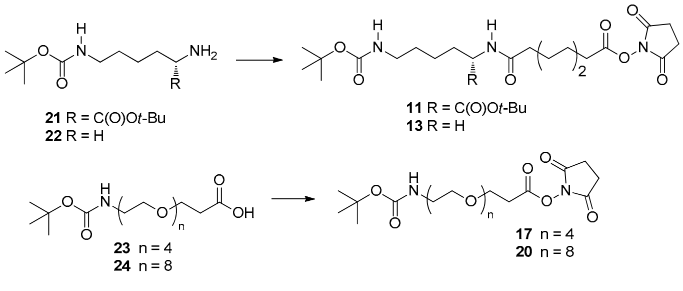

Scheme S1. Reagent and conditions: (a) CH_2Cl_2, triethylamine, suberic acid bis-N-hydroxysuccinimide ester, rt, 2h; (b) CH_2Cl_2, N-hydroxysuccinimide, dicyclohexylcarbodiimide, rt, overnight.

(*S*)-2,5-dioxopyrrolidin-1-yl 8-((1-(tert-butoxy)-6-((tert-butoxycarbonyl)amino)-1-oxohexan-2-yl)amino)-8-oxooctanoate, **(11)**: A solution of H-Lys(Boc)-O*t*Bu·HCl (**21**) (0.2 g, 0.59 mmol) and triethylamine (0.15 mL, 1.06 mmol) in CH_2Cl_2 (6 mL) was added dropwise to a stirred solution of suberic acid bis-(*N*-hydroxysuccinimide ester) (DSS, 0.7 g, 1.90 mmol) in 10 mL CH_2Cl_2, at room temperature. After 2 h, the solvent was evaporated and the crude material was purified on a silica gel gravity column using a gradient from 100% CH_2Cl_2 to CH_2Cl_2/CH_3CN 1:1 to afford 0.27 g (82%) of compound **11**. ESI-Mass calcd. for $C_{27}H_{45}N_3O_9Na$ $[M+Na]^+$ 578.3, found 578.3.

2,5-dioxopyrrolidin-1-yl 2,2-dimethyl-4-oxo-3,8,11,14,17,20,23,26,29-nonaoxa-5-azadotriacontan-32-oate, **(20)**: To a solution of *t*-Boc-*N*-amido-PEG$_8$-acid (**24**) (0.25 g, 0.46 mmol) and *N*-hydroxysuccinimide (0.053 g, 0.046 mmol) in CH_2Cl_2 (4 mL) was added dicyclohexylcarbodiimide (0.1 g, 0.048 mmol). After stirring at room temperature overnight, the mixture was filtered and the filtrate was evaporated to give compound **20**. ESI-Mass calcd for $C_{28}H_{50}N_2O_{14}Na$ $[M+Na]^+$661.3, found 661.3.

2,5-dioxopyrrolidin-1-yl 8-((5-((tert-butoxycarbonyl)amino)pentyl)amino)-8-oxooctanoate, **13**: A solution of Boc-1,5-diaminopentane, (**22**) (0.050 g, 0.25 mmol) and triethylamine (0.1 mL, 0.72 mmol) in CH_2Cl_2 (2 mL) was added dropwise to a solution of suberic acid bis-(*N*-hydroxysuccinimide ester) (DSS, 0.23 g, 0.62 mmol) in 3 mL CH_2Cl_2, under mild stirring at room temperature. After 2 h, the solvent was evaporated and the residue was purified on a silica gel column using a gradient from 100% CH_2Cl_2 to CH_2Cl_2/CH_3CN 1:1 to afford 0.072 g (64%) of compound **13**. ESI-Mass calcd for $C_{22}H_{38}N_3O_7$ $[M+H]^+$ 456.3, found 456.3.

2,5-dioxopyrrolidin-1-yl 2,2-dimethyl-4-oxo-3,8,11,14,17-pentaoxa-5-azaicosan-20-oate,***17:*** A solution of Boc-15-amino-4,7,10,1-tetraoxapentadecanoic acid, (**23**) (0.365g g, 1 mmol) and *N*-hydroxysuccinimide (0.115 g, 1 mmol) in CH_2Cl_2 (4 mL) was added dicyclohexylcarbodiimide

(0.206 g, 1mmol). After stirring at room temperature overnight, the mixture was filtered and the filtrate was evaporated to give compound **17** which was used without further purification.
